# Comparison of bicarbonate Ringer’s solution with lactated Ringer’s solution among postoperative outcomes in patients with laparoscopic right hemihepatectomy: a single-centre randomised controlled trial

**DOI:** 10.1186/s12871-024-02529-2

**Published:** 2024-04-22

**Authors:** Jie Song, Yingying Liu, Yun Li, Xiaoci Huang, Muchun Zhang, Xiaofeng Liu, Xianwen Hu

**Affiliations:** 1 Department of Anesthesiology, The Second Affiliated Hospital of Anhui Medical University, Hefei City, Anhui Province China; 2 Key Laboratory of Anesthesiology and Perioperative Medicine of Anhui Higher Education Institutes, Anhui Medical University, Hefei City, Anhui Province China; 3 Department of Anesthesiology, Children’s Hospital of Nanjing Medical University, Nanjing City, Jiangsu Province China

**Keywords:** Hyperlactatemia, Lactated Ringer’s solution, Bicarbonate Ringer’s solution, Lactic acid concentration

## Abstract

**Abstract:**

The study was aimed to investigate the positive impact of bicarbonate Ringer’s solution on postoperative outcomes in patients who underwent laparoscopic right hemihepatectomy. Patients in the two groups were infused with lactated Ringer’s solution (LRS, *n* = 38) and the bicarbonate Ringer’s solution (BRS, *n* = 38) at a rate of 5 ml·kg^–1^·h^–1^. The stroke volume was monitored and 200 ml of hydroxyethyl starch with 130/0.4 sodium chloride injection (Hes) of a bolus was given in the first 5–10 min. The main outcome was to test lactic acid (LAC) concentration before and after surgery. The concentrations of LAC in the LRS group were higher than in the BRS group at 2 h after operation began, at the end of the operation and 2 h after the operation. Overall, the parameters including pH, base excess (BE), HCO_3_^−^, aspartate transaminase (AST) and alanine transaminase (ALT) were improved. The values of bilirubin in the LRS group were higher and albumin were lower than in the BRS group at post-operation 1st and 2nd day (*P*<0.05). The time of prothrombin time (PT) and activated partial thromboplastin time (APTT) in the LRS group were longer than that in the BRS group at post-operation 1st and 2nd day (*P*<0.05). Likewise, the concentrations of Mg^2+^, Na^+^ and K^+^ also varied significantly. The length of hospital was reduced, and the incidence of premature ventricular contractions (*P* = 0.042) and total complications (*P* = 0.016) were lower in group BRS.

**Trial registration:**

The study was registered at clinicalTrials.gov with the number ChiCTR2000038077 on 09/09/2020.

**Supplementary Information:**

The online version contains supplementary material available at 10.1186/s12871-024-02529-2.

## Introduction

Lactic acid (LAC) concentration may be increased after hepatectomy [1]. It suggests that the liver blood flow and the liver’s ability to metabolise LAC may be affected after surgery [2]. According to a prior study, patients having hepatectomy procedures may have increased LAC rates of up to 71.4% [1]. Higher LAC amount is independently connected with increased risks of morbidity and mortality after hepatectomy, leading to extending the hospital stay and elevating the risk of infection [1, 3].

Cirrhosis, Charlson comorbidity index, major resections, procedure time, blood loss and transfusion, cumulative pringle time, pre-operative diabetes mellitus and type of fluids were associated with lactate level after hepatectomy [1, 4–6]. Shin et al. found that intraoperative infusion of lactic acid-free crystalloid solution may have a great advantage over LRS solution [5]. Patients undergoing liver resection who use LRS experience an increase in the total amount of lactate, resulting in an increased LAC concentration [7].

Sodium bicarbonated Ringer’s (BRS) solution is a new type of crystalloid solution, which is the closest equilibrium crystalloid to the composition of extracellular fluid [8]. Studies have shown that BRS has a significant effect on replenishing circulating blood volume, maintaining Mg^2+^ concentration, reducing the LAC level and correcting metabolic acidosis [9]. But the effect of BRS on patients undergoing laparoscopic right hemihepatectomy still remains unknown. Hence, we designed a randomised controlled trial to compare the outcomes of BRS and LRS on LAC and postoperative outcomes in patients following laparoscopic right hemihepatectomy, with the aim to provide clinical reference.

## Materials and methods

### Ethics

This randomised clinical study was approved by the Ethics Committee for Clinical Trials of the Second Affiliated Hospital of Anhui Medical University (Chairperson Prof Jing Zhang) [SL-YX2020-051] and registered at clinicalTrials.gov with the number ChiCTR2000038077 on 09/09/2020. Before the patients were enrolled, all patients signed an informed consent.

### Participants

This randomised clinical study was designed and conducted according to the guidelines of the Consolidated Standards of Reporting Trials (CONSORT). We recruited 80 hepatic carcinoma patients who were scheduled for elective laparoscopic right hemihepatectomy in the Second Affiliated Hospital of Anhui Medical University between October 2020 and August 2021. In this study, patients aged 45–60 years old with a body mass index (BMI) of 18–26 kg m^− 2^ and an American Society of Anaesthesiologists (ASA) II or III status were enrolled. Exclusion criteria prior to randomisation included: (1) taking drugs that damage liver function within half a year before admission or with liver function child-pugh score of > 6 points; (2) having serious chronic diseases of the heart, lung, kidney or other organs; (3) failing to maintain normal vital signs after repeated intraoperative use of vasoactive drugs and infusion of large amounts of fluids and blood products; (4) the operation time being less than 4 h; (5) participate in other treatments or refusal to participate. Reasons for exclusion following randomisation included protocol violations and change in surgical procedures.

Once consent was obtained, an anaesthesia nurse who did not participate in this study divided the participants into two groups based on a random assignment sequence using online random list generation to perform randomisation at a ratio of 1:1. The details of each patient’s infusion method and intraoperative anaesthesia management were stored in an opaque sealed envelope, which was only opened by the researcher when the patient entered the operating room. All participants, data collectors and statisticians had no knowledge of group assignments.

### Anaesthesia procedure

All patients were fasting for 8 h and forbidden to drink for 4 h before the procedure. Then they received midazolam 0.2 mg kg^–1^ after arriving in the operating room. The peripheral venous access was established, and the right internal jugular vein and right radial artery were punctured. The heart rate (HR), oxygen saturation (SpO_2_), end-tidal carbon dioxide (PTCO_2_), arterial blood pressure, mean arterial pressure (MAP) and central venous pressure were monitored continuously by a monitor (model: infinityC700, Germany, Draegerwerk AG & COKGaA). For BIS monitoring, a disposable BIS sensor (BISTM sensor; America, Covidien IIc Co.) was stuck to the forehead after alcohol swabbing the skin. The stroke volume (SV) and cardiac index (CI) was monitored using pulse contouring (Vigileo 03.06, Edwards Lifesciences, Irvine, CA, USA) and special pressure sensors (FloTrac system, Edwards Lifesciences). Before intubation, 100% oxygen was supplied by the mask. General anesthesia was induced with etomidate 0.2 mg kg^–1^, sufentanil 0.5 µg kg^–1^ and cisatracurium 0.2 mg kg^–1^. After tracheal intubation, all patients underwent mechanical ventilation with a tidal volume of 8 ml kg^–1^, inhaling a 60 and 40% mixture of oxygen and air, with an inhalation-expiration ratio of 1:2 with a goal to achieve PETCO_2_ 35–45 mm Hg and SpO_2_> 95%. Anesthesia was maintained with propofol (4–8 mg kg^–1^ h^–1^), remifentanyl (0.1–0.3 µg kg^–1^ min^–1^), cistracurium (0.1 − 0.2 mg kg^–1^ h^–1^) and sevoflurane (1−2%), to maintain BIS values between 40 − 60. The patient’s body temperature was monitored by a nasal thermometer (Shenzhen, Mecun, Medical) and found maintained at 36.0 − 37.0 ^o^C. Target for intraoperative mean arterial pressure, heart rate and haemoglobin were ≥ 80% of baseline, between 60 –100 bpm and ≥ 8 g dL^–1^ respectively. If the mean arterial pressure dropped by more than 30% from the baseline, a single IV bolus of phenylephrine 40–80 µg was administered. If the blood pressure continued to decrease during the operation or when the hilus hepatis was blocked and if there was no response to the fluid bolus, the phenylephrine continuously pumped [10]. Sevoflurane and cisatracurium were discontinued and sufentanil 10 µg was given 30 min before the end of the operation. Remifentanyl and propofol infusion were stopped at the end of surgery. After surgery, the patients were sent to AICU. When the patients had spontaneous breathing, they were given atropine 0.5 mg and neostigmine 1 mg to antagonise the residual muscle relaxant effect. After reaching the conventional extubation indications, the tracheal catheter was removed, and the continuous mask or nasal catheter was given oxygen inhalation and vital signs were monitored. When the Steward Resuscitation score was found above 4, all patients were transferred to the ward. All patients received patient controlled IV analgesia (PCIA) after operation with the formulation of sufentanil 2.5 µg kg^–1^ with metoclopramide 20 mg diluted to 100 ml. The first dose of PCIA treatment was 2 ml, the background dose was 2 ml h^–1^, PCIA dose was 0.5 ml and the lockout time was 15 min.

### Intraoperative fluid management

Patients in the two groups were infused with LRS and BRS at a rate of 5 ml kg^–1^ h^–1^. BRS differs from LRS as it does not have lactate and it contains 28 mmol L^–1^ HCO_3_^−^ [8]. The stroke volume was monitored and 200 ml of hydroxyethyl starch with 130/0.4 sodium chloride injection (Hes) of a bolus was given in the first 5–10 min. The fluid bolus was repeatedly used in patients with response (stroke volume increased by 10%) until the Frank-Starling relationship reached the platform stage. Additional bolus was given only when stroke volume dropped by >10% below the plateau value [11].

### Outcome measures

The aim was to test LAC concentration at 2 h after the operation began, at the end of the operation, at 2 h after the operation and 1- and 2-days post operation using a GEM Premier 3500 blood gas analyser (United States of America, Werfen co). BE, HCO_3_^−^ and pH were recorded at the same time. Intraoperative crystalloid volume,  Hes volume, total fluid volume, duration of pneumoperitoneum, hepatic portal occlusion, operation **and the hospital stay after operation**, number of blood tansfusion,urine volume, estimated blood loss volumeand dose of phenylephrine agents were measured during the operation. Mg^2+^, Na^+^ and K^+^ concentration and bilirubin, albumin, hemoglobin values were recorded at 1- and 2- days post operation along with PT and APTT. Liver function was checked at 1-, 3- and 7-days post operation and postoperative complications (including postoperative premature atrial contractions, premature ventricular contractions, liver failure, liver abscess, biliary leakage, biloma, renal failure wound infection, ascites, hemoperitoneum, intra-abdominal infection, venous thrombosis, total incidence of complications and 90-days mortality rate). Total incidence of complications was calculated as (number of patients with complications/total number of patients) × 100%.

### Sample size

The sample size was evaluated with G*power software. The mean LAC concentration at 2 h after the operation began was used as the indicator for the efficacy evaluation in the calculation of the sample size. Results from our preliminary experiments showed that the mean LAC concentration was 1.28 ± 0.40 after BRS infusion and 0.97 ± 0.27 after LRS infusion. Using 5% bilateral alpha levels, 80% power and 10% exit rates, a minimum of 35 subjects per group was required.

### Statistical analysis

SPSS version 21.0 was used to conduct statistical analysis (SPSS Inc., Chicago, IL, USA). The Kolmogorov-Smirnov test and the Levene test were used to determine whether the measurement data had a normal distribution and whether the variance was homogeneous. The Shapiro-Wilk test was used to determine data normality.

Mean and standard deviation (SD) were used to depict data with a normal distribution. Student’s t-test was employed to compare the groups. Repeated measurement analysis of variance (ANOVA) was used to assess the data gathered at various time points. The classification variables were represented by numbers (%). Categorical variables such as American Society of Anaesthesiologist (ASA) classification, sex, number of pre-operative hypertension, number of blood transfusions and the prevalence of postoperative difficulties were assessed using the χ^2^ test or Fisher’s exact tests for smaller events (< 5). Age, body mass index (BMI), pneumoperitoneum duration, hepatic portal occusion and operation duration,number of blood tansfusion, estimated blood loss volume, urine volume, dose of phenylephrine agents, hospital stay following surgery, AST and ALT at preoperative and 1-, 3- and 7-days post operation, the time of PT and APTT, bilirubin, albumin, haemoglobin and Mg^2+^, Na^+^ and K^+^ concentrations at pre-operative and 1- and 2-days post operation were all compared between the two groups using an independent sample t-test. The data of LAC concentration, BE, pH, HCO_3_^−^, AST and ALT, the time of PT, APTT, bilirubin, albumin, haemoglobin, Mg^2+^ Na^+^ and K^+^ concentrations values were analysed using repeated measurement analysis of variance. *P* value less than 0.05 considered statistically significant.

## Results

### Patient characteristics

**Fig. 1:**
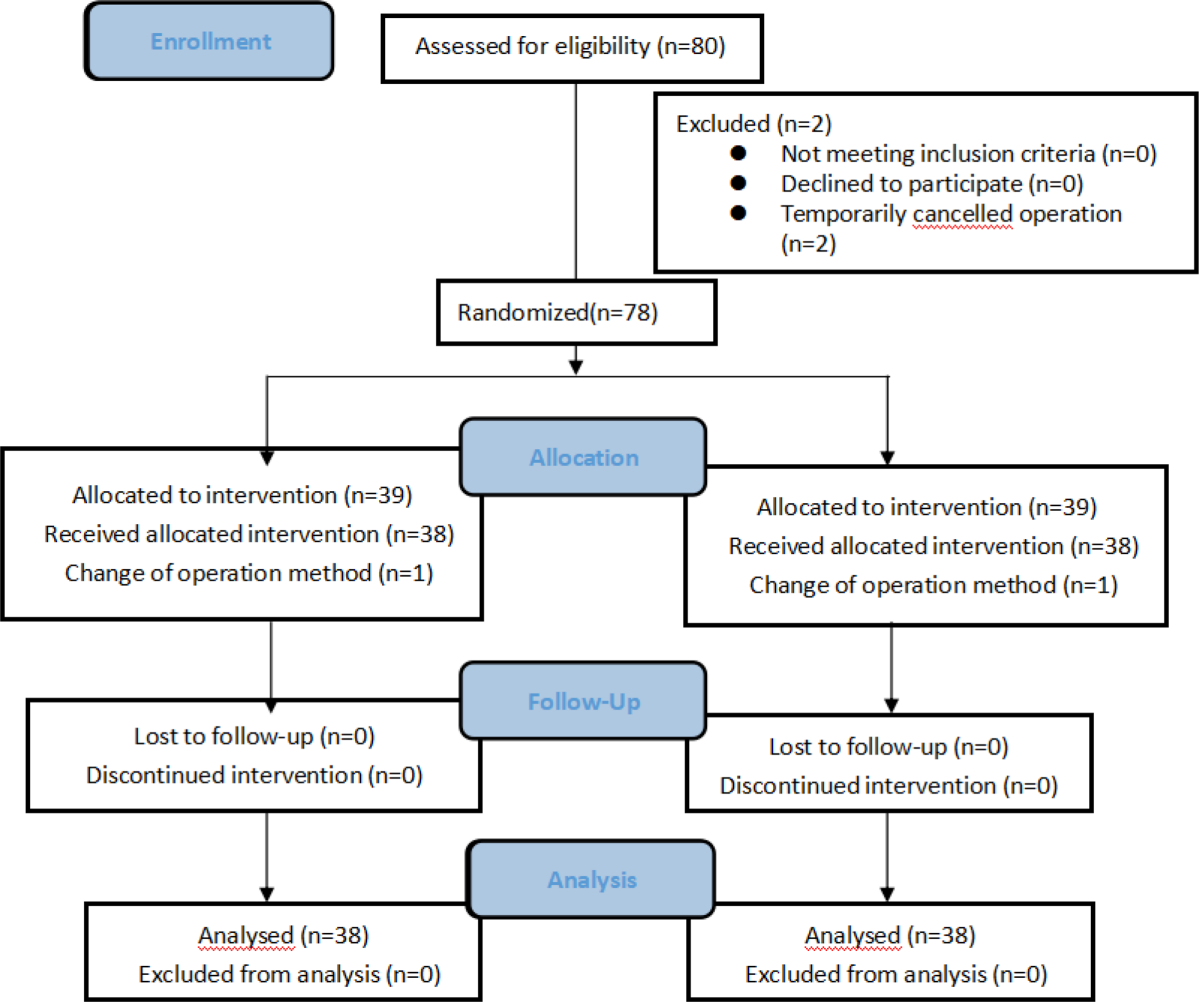
CONSORT flow diagram of the study

A total of 80 patients with laparoscopic right hepatectomy were evaluated, of which 2 patients temporarily cancelled the operation and 2 patients converted to open surgical techniques. To conclude the study, 76 patients were involved, with 38 cases in the BRS and 38 cases in the LRS groups. The CONSORT flow diagram of patient selection is shown in Fig. 1. There was no significant difference in baseline characteristics between the two groups (Table 1).

**Table 1:** Demographic characteristics of the study patients

	Group LRS (*n* = 38)	Group BRS (*n* = 38)	P-value
Age (y)	54.6 ± 5.4	55.1 ± 5.6	0.738
BMI (kg/m^2^)	21.9 ± 1.5	21.5 ± 0.9	0.139
**ASA physical status**			
II	20(53)	22(58)	0.645
III	18(47)	16(42)	0.645
HBV (+)	32	30	0.497
Hypertension	15(39)	13(34)	0.589
**Grade of hypertension (n)**			
I	9(24)	8(21)	0.574
II	6(16)	7(18)	0.877

Value expressed as Mean ± SD or number (%); ASA = American Society of Anesthesiologists; BMI = body mass index

### Primary outcome

The LAC values had no significant difference at baseline (Pre) or 2 days after the operation (POD2). Compared with the baseline value, LAC increased 2 h after the operation began (2 h) in group LRS (*P* <0.05) while there were no significant changes in group BRS (*P* >0.05). Lac concentration increased at the end of operation (end) and continued to rise to a peak at 2 h post operation (2 h-POD) in the two groups, while it still remained above the baseline at 1 day post operation (POD1) (*P* <0.05). LAC values remained higher in LRS group than in BRS from operative period to the day after surgery (2 h to 2 h-POD) (*P* <0.05) (Fi﻿g. 2).

**Fig. 2:**
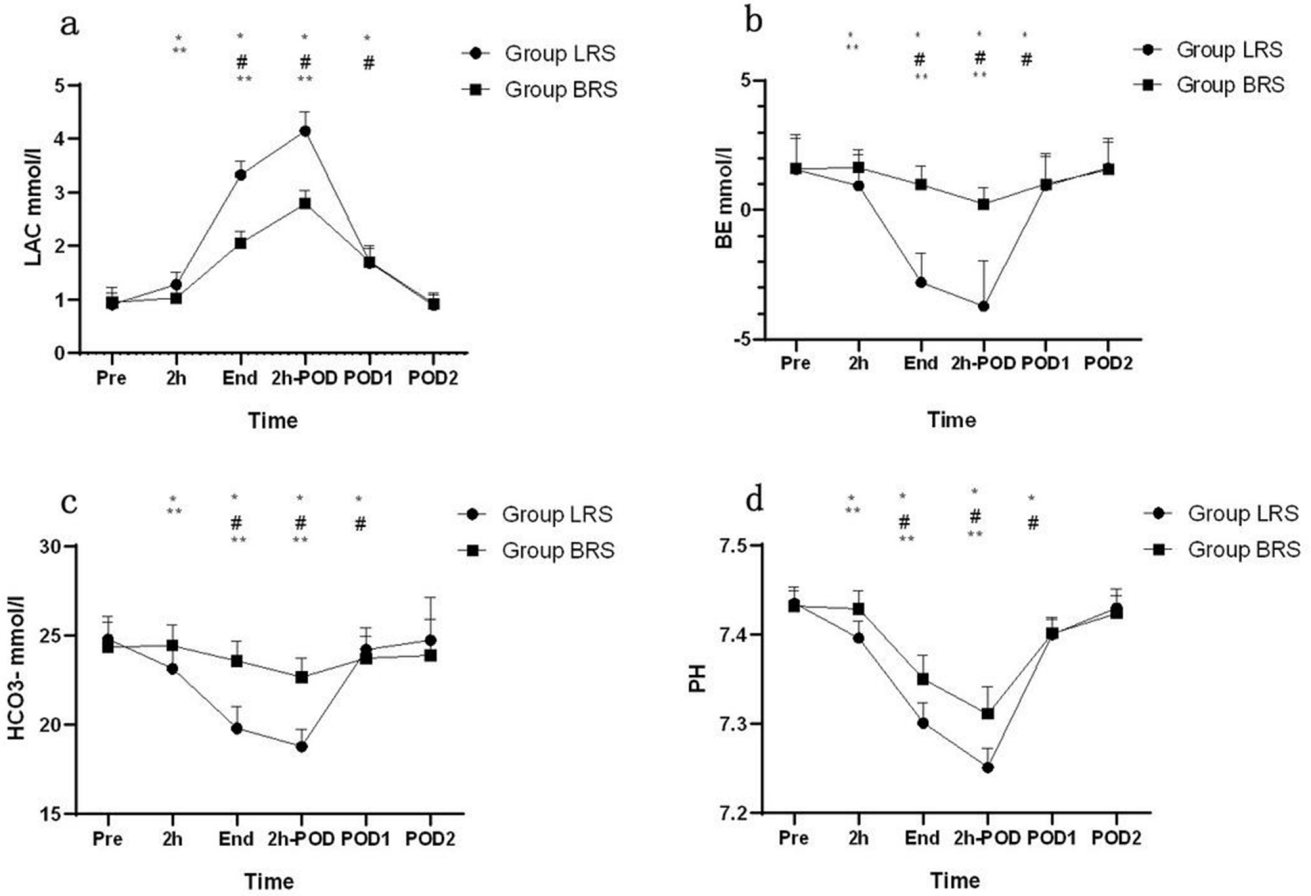
Changes of LAC, BE, HCO_3_^−^ and pH during and after Laparoscopic right hemiheparatectomy. Pre, preoperative; 2h,2h after the operation began; End, the end of operation; 2h-POD, 2h postoperatively; POD1, 1st postoperative days; POD2, 2nd postoperative days Lac (**a**), BE (**b**), HCO_3_^-^ (**c**) and PH (**d**) levels between group BRS and group LRS.Data are presented as Mean ± SDs. Compared with Pre in Group LRS, *P＜ 0.05; Compared with Pre in Group BRS, ^#^P＜ 0.05; Compared with Group LRS, **P＜ 0.05

### Secondary outcomes

The values for pH, BE and HCO_3_^−^ decreased at 2 h after the opretiaon began, the end of operation and 1 day post operation when compared with pre-operative values in the two groups (*P* < 0.05). At 2 h after the operation began, pH, BE and HCO_3_^−^ decreased in group LRS (*P* <0.05) while there were no significant changes in BRS group (*P* >0.05). At 2 h after the operation began, the end of operation and 2 h post operation, pH, BE and HCO_3_^−^ remained lower in LRS group than in BRS (*P* <0.05). At 2 h post operation, pH, BE, HCO_3_^−^ were reached to the lowest values in both the groups (Fig. 2).

No differences in duration of pneumoperitoneum, hepatic portal occlusion and surgery, total intraoperative fluid volume, intraoperative crystalloid solution volume, intraoperative HES volume, number of blood tansfusion, estimated blood loss volume, urine volume and the use of phenylephrine agents were found between the two groups (*P* >0.05). Compared with the LRS group, the hospital stay after operation were significantly shortened in group BRS (*P*<0.05) (Table 2).

**Table 2:** Surgical data with outcomes

	Group LRS (*n* = 38)	Group BRS (*n* = 38)	P-value
Duration of pneumoperitoneum (min)	293.1 ± 44.7	285.9 ± 48.2	0.501
Hepatic portal occlusion time (min)	50.0 ± 8.7	49.0 ± 7.9	0.654
Duration of operation (min)	323.0 ± 45.7	316.3 ± 48.9	0.538
Total intraoperative fluid volume (ml)	2513.2 ± 244.9	2485.5 ± 257.6	0.633
Intraoperative crystalloid solution volume (ml)	1906.6 ± 174.8	1847.4 ± 190.0	0.162
Intraoperative HES volume (ml)	638.2 ± 111.2	610.5 ± 110.4	0.280
Number of blood transfusion (n)	0	0	1.000
Estimated blood loss volume (ml)	317.1 ± 80.0	290.8 ± 81.3	0.159
Urine volume (ml)	596.1 ± 110.5	607.9 ± 130.2	0.670
Dose of phenylephrine agents (ug)	237.4 ± 53.3	233.7 ± 53.8	0.765
The hospital stay after operation (days)	12.9 ± 1.4	10.8 ± 1.5*	0.000

Duration of pneumoperitoneum refers to from the beginning of pneumoperitoneum to the end of pneumoperitoneum. Hepatic portal occlusion time refers to total time of hilus hepati blocked. Duration of surgery refers to from surgery begins to end *P＜0.05 vs. Group LRS

**Fig. 3:**
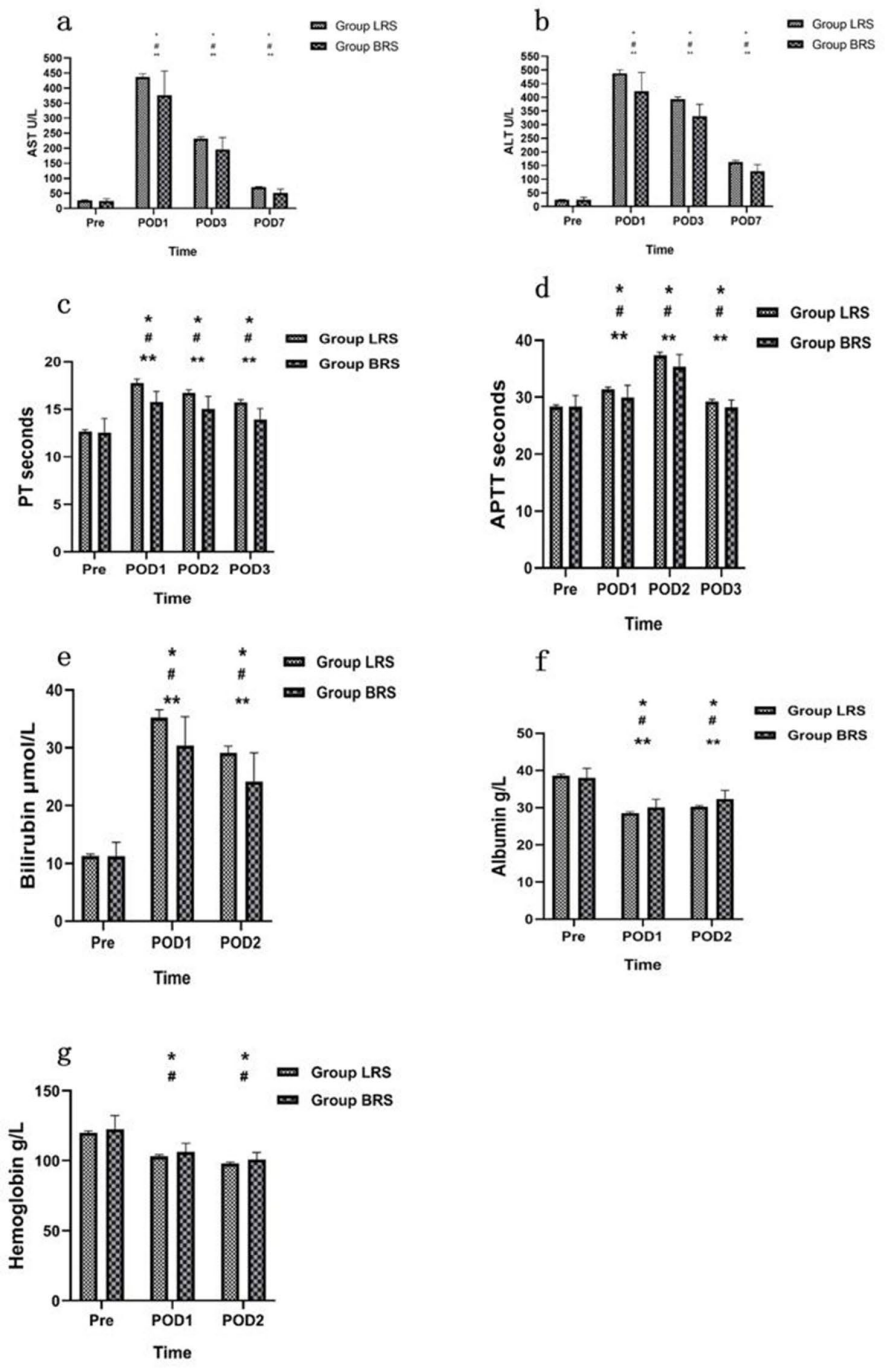
Postoperatively AST, ALT, PT, APTT, bilirubin, albumin, haemoglobin concentrations outcomes. Pre, preoperative; POD1, 1st postoperative days; POD2, 2nd postoperative days; POD3, 3rd postoperative days; POD7, 7th postoperative days. AST (**a**), ALT (**b**), PT (**c**), APTT (**d**), Bilirubin (**e**), Albumin (**f**), Haemoglobin (**g**) levels between group BRS and group LRS; Data are presented as Mean ± SD. Compared with Pre in Group LRS, *P ＜ 0.05; Compared with Pre in Group BRS, ^#^P＜0.05; Compared with Group LRS, **P＜0.05

AST and ALT were elevated at 1st, 3rd and 7th day post operation in two groups (*P* <0.05). Compared with the group LRS, AST and ALT values were lower at 1st, 3rd and 7th day post operation in group BRS (*P* <0.05). The levels of PT, APTT, bilirubin were elevated, while albumin level was decreased at 1st and 2nd day post operation in two groups (*P* <0.05). Compared with values in group LRS, PT, APTT and bilirubin were lower, and albumin was higher at 1st and 2nd day post operation in the BRS group (*P* <0.05). The values for haemoglobin were lower than pre-operation in the two groups on the 1st and 2nd day post operation (*P* <0.05). But there were no significant differences in haemoglobin concentrations between the two groups *(P* >0.05) (Fig. 3).

**Fig. 4:**
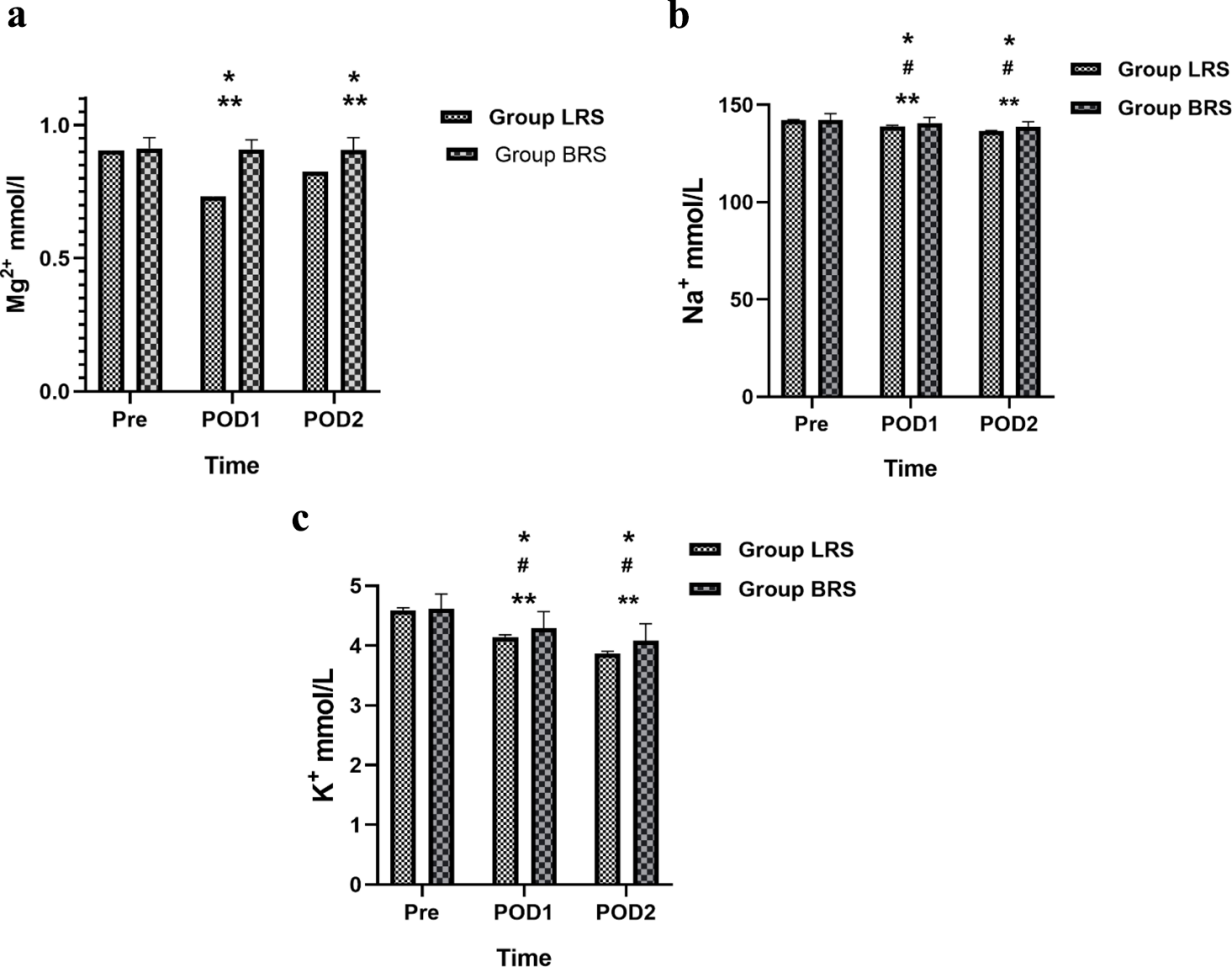
Postoperatively Mg^2+^, Na^+^ and K^+^ concentrations outcomes. Pre, preoperative, POD1, 1st postoperative days; POD2, 2nd postoperative days. Mg^2+^ (**a**), Na^+^ (**b**), K^+^ (**c**) concentrations values between group BRS and group LRS; Data are presented as Mean ± SD. Compared with Pre in Group LRS, *P＜ 0.05; Compared with Pre in Group BRS, ^#^P＜0.05; Compared with Group LRS, **P＜ 0.05

Na^+^ and K^+^ concentrations were dropped at 1st and 2nd day post operation in two groups and Mg^2+^ concentrations were dropped at 1st and 2nd day post operation in LRS group (*P* <0.05). Compared with values in group LRS, Mg^2+^, Na^+^ and K^+^ concentrations were higher at 1st and 2nd day post operation in group BRS (*P* <0.05) (Fig. 4).

### Postoperative complications

The incidence of premature ventricular contractions and total incidence of complications were higher in the group LRS than those in group BRS (*P* <0.05). There was no statistical significance in premature atrial contractions, liver failure, liver abscess, biliary leakage, biloma, renal failure wound infection, ascites, hemoperitoneum, intra-abdominal infection, venous thrombosis and 90-day mortality rate between the two groups (*P* >0.05) (Table S1).

## Discussion

Normal saline has been used historically as an intraoperative fluid. however, its use has been linked with some complications [8]. Hence, some alternatives were developed, particularly, crystalloid solutions. In this context, A retrospective study conducted in a Canadian tertiary hospital to compare the impact of normal saline, balanced crystalloids and combination of both to vascular surgery patients showed a better outcome of balanced crystalloids in terms of ventilator requirements and mortality [12]. In contrast, a recent study reported an association of balanced crystalloid solution with a higher mortality rate in patients having out of hospital cardiac arrest, than the normal saline. However, the latter was found to be associated with complications in all the study population of cardiogenic shock patients [13]. While comparing normal saline, Ringer’s solution and Plasmalyte Salinero et al. argued that although Ringer’s solution and Plasmalyte are better than normal saline, however, considering their higher potassium content, they should be administered carefully to the patients with hyperkalemia. Nonetheless, the literature they reviewed presented Plasmalyte as a better substituent to Ringer’s solution owing to its near-to-plasma composition [14]. Considering these contrasting observations, there was a need to understand the precise impact of BRS and LRS.

Therefore, the primary goal of the current study was to investigate LAC concentration in the patients’ groups receiving either LRS or BRS during and after surgery. Previously, in mice model, bicarbonate ringer’s solution appeared as more effective during resuscitation of haemorrhagic shock and could protect various organs as compared to normal saline [8]. Bicarbonate in BRS is similar to the body’s buffering system and can provide an alkalinizing effect when used as a resuscitating agent. It can address the problem the body faces during metabolic acidosis. The other electrolytes including sodium ion, potassium ion, calcium ion, magnesium ion and chloride ion also mimic the natural chemistry of body fluids [8].

The data of this first prospective randomised study found that LRS could slightly increase lactic acid concentration from 2 h intraoperative to the day after the operation and accordingly worsen postoperative outcomes in laparoscopic right hemihepatectomy. The reasons for the elevated lactic acid concentration in laparoscopic right hemihepatectomy can be attributed to the fact that the liver is the main site of LAC metabolism that can clear 70% of LAC by converting it into glycogen [15]. Therefore, most patients experience transient hepatic insufficiency during major hepatectomy which may lead to hyperlactatemia. Moreover, special surgical and anaesthetic techniques employed in hepatectomy, such as long periods of carbon dioxide pneumoperitoneum, the length of the operation, liver parenchyma injury, hepatic portal occlusion techniques, infusing lactate-containing solution and blood loss may also cause hyperlactatemia condition [1, 4, 15, 16]. Also, the intraoperative release of adrenaline and amylin which has been found to stimulate LAC production [17]. Therefore, patients receiving right hemihepatectomy treatment suffer with insufficient lactate metabolism and increased lactate production, which lead to increased postoperative lactate levels.

Currently, LRS and normal saline are the most commonly used intra-operative crystalloid solutions [14]. Yet, harmful effects of hyperchloric acidosis after normal saline infusion to liver donors have been reported [18]. LRS contains 28 mmol L^–1^ of lactate with pH 6.5 and has been selected as the preferred perioperative extracellular fluid replacement [19]. However, the metabolism of LAC to bicarbonate mainly depends on liver function. Therefore, the addition of exogenous LAC may increase its concentration when the liver function is impaired after hepatectomy [20]. In contrast, BRS is a balanced crystalloid solution containing physiological concentrations of bicarbonate and electrolyte ions [8]. Bicarbonate ions in BRS could be directly combined with surplus hydrogen ions in the body without liver metabolism, correcting metabolic acidosis without increasing the burden on the liver [8]. Studies have shown that BRS could significantly correct acid-base imbalance compared with other crystalloid solutions [21, 22].

The hypothesis tested in this study was based on a previous report where a lactate-free crystalloid solution showed better results than the LRS solution in LAC and BE during hepatectomy [5]. In subsequent clinical trials, BRS improved LAC metabolism and maintained acid-base balance in individuals with extreme multiple traumas and traumatic shock and reduced the risk of related complications [9]. While earlier, Satoh et al. used rabbit model and found that BRS was the most effective perioperative solution for the treatment of metabolic acidosis and plasma electrolyte balance in partially hepatectomised animals when compared with other crystalloid solutions [22]. Recently, Wang et al. investigated the effectiveness of BRS in traumatised adult male New Zealand rabbits and found that the BRS could reduce the extent of elevated LAC concentrations and markedly correct the acid-base imbalance in hemorrhagic shocked rabbits [8]. In our study, pH, BE and HCO_3_^−^ levels decreased with an increase in concentration in group LRS at 2 h after the operation began, while no significant differences were observed in the BRS group. Moreover, in the LRS group, LAC remained elevated until the end of operation and 2 h post-operation, while in the BRS group it was at lower side. It indicates that the administration of BRS can correct intraoperative and postoperative metabolic acidosis in the patients encountering laparoscopic right hemihepatectomy.

LAC had a strong correlation with the clinical outcome of critically ill patients and was a key indicator for diagnosing shock or “resuscitation” [23–25]. According to a systematic review, LAC is a useful predictor of postoperative outcome after hepatectomy, particularly when measured early after surgery [1]. They discovered that LAC levels greater than 3.7 mmol L^–1^ in the early stages of recovery are associated with mortality. Wiggans et al. discovered a link between early LAC after hepatectomy and postoperative peak bilirubin, renal insufficiency, hospitalisation time and 90-day mortality [6]. In addition, a study showed that regardless of the patient’s liver pathology, the highest level of lactate during surgery may be a predictor of postoperative infection complications [26]. In our study, the hospital stay after operation was significantly shortened, the incidence of premature ventricular contractions and total incidence of complications were lower in group BRS than those in the LRS group, presenting BRS could promote patients’ postoperative outcomes.

While considering other parameters, the levels of AST and ALT were higher on the 1st, 3rd and 7th days post operation in LRS than in the group BRS. Likewise, Bilirubin, PT and APTT levels were higher and albumin was lower at 1st and 2nd day post-operation in LRS compared to the group BRS. This finding should be taken in the light of mitochondrial function after hepatectomy [27] as it is significantly correlated with arterial LAC value on the day of surgery, transaminase on the first day after surgery and total bilirubin on the fifth day [26]. Hence, the higher lactate in the LRS group may have lower mitochondrial functions, which led to poor postoperative liver function. A study by Wang et al. found that BRS improved the hepatocyte apoptosis caused by shock and had a certain protective effect on the liver [8].

It is pertinent to record that Mg^2+^, Na^+^ and K^+^concentration often decreased in hepatic insufficiency caused by hepatectomy [28–30]. This is associated with decreased albumin as well as multiple disordered hormone secretion [28, 31, 32]. When liver function is impaired, aldosterone and antidiuretic hormone inactivation is reduced that leads to increased potassium excretion and dilutive hyponatremia [32, 33]. Many studies have linked higher rates of complications and mortality with inadequate Mg^2+^, Na^+^ and K^+^ ions [34–37]. Our results found that compared with values in the group LRS, Mg^2+^, Na^+^ and K^+^ concentrations were higher at 1st and 2nd day post-operation in the group BRS and Mg^2+^ concentrations only dropped at 1st and 2nd day post-operation in LRS group which exhibited that the Mg^2+^ contained in the BRS was complementary to the intraoperative Mg^2+^ loss. Meanwhile, the protective effect of BRS on the liver was evident as it helped to maintain better electrolyte balance indicating reduced hormonal disturbances after hepatectomy [8]. BRS administration also assisted to decrease the incidence of premature ventricular contraction that could be linked with improved electrolyte balance [34, 38, 39] leading to lower efficiency of the Na^+^/K^+^ ATPase system that also affected membrane potential [40, 41].

While concluding the findings of this study, certain limitations cannot be ignored. Firstly, only two crystal balanced liquids, LRS and BRS were compared. It would be more meaningful to design a multi-arm study including acetated Ringer’s solution, sodium bicarbonate solution and normal saline. Secondly, there was no long-term follow-up and BRS was only applied briefly and during the intraoperative phase. Thirdly, the study lacked measures of blood immune indexes, such as IL-1, IL-6, TNF-α and IGF-I. The extension of our study to other groups was further constrained because it used a small sample size of a single centre with detailed inclusion and exclusion criteria. Thus, more indicators, large sample size and multicentre studies are required to compare the effects of LRS and BRS on LAC metabolism and postoperative outcomes in laparoscopic right hemihepatectomy.

## Conclusions

The positive impacts of BRS administration were evident by considering the LAC concentration and electrolyte balance. BRS assisted to reduce the level of elevated LAC concentrations, markedly corrected metabolic acidosis and reduced the incidence of complications. Hence, BRS appeared as a good substitute crystalloid for patients undergoing hemihepatectomy.

### Electronic supplementary material

Below is the link to the electronic supplementary material.

Supplementary Material 1

## Data Availability

The data sets used and/or analysed during the study are available from the corresponding author on reasonable request. The email of corresponding author is huxianwen001@outlook.com.

## Abbreviations

LAC: lactic acid
LRS: lactated Ringer’s solution
BRS: Sodium bicarbonated Ringer’s
BE: base excess
CONSORT: Consolidated Standards of Reporting Trials
ASA: American Society of Anesthesiologists
HR: heart rate
SpO_2_: oxygen saturation
PTCO_2_: end-tidal carbon dioxide
MAP: mean arterial pressure
SV: stroke volume
CI: cardiac index
PCIA: patient-controlled IV analgesia
Hes: hydroxyethyl starch with 130/0.4 sodium chloride injection
SD: standard deviation
ANOVA: analysis of variance
AST: aspartate aminotransferase
ALT: alanine aminotransferase
PT: prothrombin time
APTT: activated partial thromboplastin time
