# Supplementary material for: Comparison of bicarbonate Ringer’s solution with lactated Ringer’s solution among postoperative outcomes in patients with laparoscopic right hemihepatectomy: a single-centre randomised controlled trial
Source: BMC Anesthesiol. 2024 Apr 22;24:152. doi: 10.1186/s12871-024-02529-2 (PMC11034129; doi:10.1186/s12871-024-02529-2)
Supplement: Supplementary file 1 — Supplementary Material 1 [file 12871_2024_2529_MOESM1_ESM.docx]

**Table S1 Complications and** [**mortality**](about:blank) **after surgery**

|  | **Group LRS**  **(n = 38)** | **Group BRS**  **(n = 38)** | **P** |
| --- | --- | --- | --- |
| Premature atrial contractions (n) | 3(8) | 2(5) | 1.000 |
| premature ventricular contractions  (n) | 8(21) | 2(5) | 0.042* |
| Liver/biliary (n） |  |  |  |
| Liver failure | 5(13) | 2(5) | 0.430 |
| Liver abscess | 3(8) | 1(3) | 0.615 |
| Biliary leakage | 4(11) | 2(5) | 0.674 |
| Biloma | 0(0) | 0(0) | 1.000 |
| Pulmonary(n) |  |  |  |
| Pleural effusion | 5(13) | 3(8) | 0.711 |
| Genitourinary(n) |  |  |  |
| Renal failure | 0(0) | 0(0) | 1.000 |
| Miscellaneous(n) |  |  |  |
| Wound infection | 3(8) | 2(5) | 1.000 |
| Ascites | 4(11) | 2(5) | 0.674 |
| Hemoperitoneum | 3(8) | 1(3) | 0.615 |
| Intra-abdominal infection | 5(13) | 2(5) | 0.430 |
| Venous thrombosis | 0(0) | 0(0) | 1.000 |
| Total incidence of complications | 47 | 21 | 0.016* |
| 90-day mortality | 3(8) | 1(3) | 0.615 |

Total incidence of complications = (number of patients with complications/total number of patients) × 100%. Compare with vs Group LRS.^⁎^P < 0.05.
